# Diaper need is associated with risk for food insecurity in a statewide sample of participants in the Special Supplemental Nutrition Program for Women, Infants, and Children (WIC)

**DOI:** 10.1016/j.pmedr.2021.101332

**Published:** 2021-02-23

**Authors:** Emily H. Belarmino, Amy Malinowski, Karen Flynn

**Affiliations:** a Department of Nutrition and Food Sciences, University of Vermont, 109 Carrigan Drive, 256 MLS Carrigan Wing, Burlington, VT 05405-0086, USA; b Vermont Department of Health, Maternal and Child Health Division, 108 Cherry Street, Burlington, VT 05402, USA

**Keywords:** Diapers, Food security, Nutrition assistance, Poverty, Children, IRB, Institutional Review Board, HFSS, Household Food Security Scale, NECTA, New England City and Town Area, SNAP, Supplemental Nutrition Assistance Program, TANF, Temporary Assistance Program for Needy Families, WIC, Special Supplemental Nutrition Program for Women, Infants, and Children

## Abstract

Diaper need is a form of material hardship that acutely affects families with young children, is not currently addressed by US antipoverty programs, and has received little public or scientific attention. This study examined the association between diaper need and risk for food insecurity in a statewide sample of participants in the Special Supplemental Nutrition Program for Women, Infants, and Children (WIC). Families enrolled in Vermont WIC in August 2019 were invited to an online survey that included questions related to diaper need and food need. Pearson’s Chi-square tests were used to examine the association between diaper need and risk for food insecurity measured by the Hunger Vital Sign Tool, and the associations between diaper need and other household factors. Follow-up questions asked those with diaper need what they do when they run out of diapers and those without diaper need how they access enough diapers. Responses to these questions were tabulated. Complete data were available for 501 households. Over half (52.3%) were at risk for food insecurity and nearly one-third (32.5%) reported diaper need. Households with diaper need were more likely to be at risk for food insecurity than those without diaper need (*p* < 0.001). Strategies to avoid running out of diapers included borrowing, stretching supplies, seeking diapers from an agency or support organization, switching to cloth or underwear, and buying on credit. Interventions that address diaper need may alleviate hardship and support health equity among families with young children.

## Highlights

• One-third of low-income families with a child in diapers report diaper need.
• Families with diaper need are more likely to be at risk for food insecurity.
• Disposable diapers represent a considerable financial burden for young families.
• Addressing diaper need may provide a tangible way to reduce food insecurity.

## 1 Introduction

In 2018, 14.3% of American households with children aged 0–5 years were food insecure at some point during the previous year, and in 6.7% of those households, at least one child experienced food insecurity (Coleman-Jensen et al., 2019). Food insecurity during early childhood has been associated with poorer general health, developmental risk, and hospitalizations (Drennen et al., 2019), and may be particularly deleterious given the long-term consequences of failing to optimize growth and brain development during early life (Cusick and Georgieff, 2016).

Poverty is a well-established risk factor for food insecurity (Coleman-Jensen et al., 2019). Among the most important federal programs in the United States aimed at reducing the effects of poverty on young children are those that provide food benefits, especially the Special Supplemental Nutrition Program for Women, Infants, and Children (WIC) and the Supplemental Nutrition Assistance Program (SNAP; formerly Food Stamps) (Pac et al., 2017). Participation in these programs reduces food insecurity (Kreider et al., 2016, Shaefer and Gutierrez, 2013). However, since households with greater levels of need tend to seek assistance from these programs, food insecurity among participating households remains high. In 2018, 47.5% of households that received SNAP benefits were food insecure, as were 36.9% of households that received WIC benefits (Coleman-Jensen et al., 2019).

For families living in poverty, the cost of diapering a child can be a source of considerable financial and emotional strain. Unlike food, diapers are not targeted by any in-kind federal assistance program and must be purchased with cash resources. Failure to provide enough diapers increases risk for diaper dermatitis (Scheinfeld, 2005) and urinary tract infections (Sugimura et al., 2009), as well as poor parental mental health (Austin and Smith, 2017, Smith et al., 2013), a mediator between poverty and child health outcomes (Ashiabi and O’Neal, 2007). Diaper need refers to lack of a sufficient supply of diapers to change children as often as needed (Smith et al., 2013). Documentation of diaper need in the United States remains limited.

Direct cash assistance to the nation’s poorest families has declined over the past thirty years and this decline has been associated with increased hardship for families with children (Shaefer et al., 2020). Recent research estimates that more than a third of US children live in households that experience material hardship and has found that different forms hardship tend to co-occur (Rodems and Shaefer, 2020). For poor families with young children, food and diaper needs may overlap (Austin and Smith, 2017, Massengale et al., 2017). However, studies linking these two forms of hardship have not utilized valid measures of food need. The aims of this study are to quantify diaper need in a statewide sample of low-income families participating in the WIC program and examine the association between diaper need and risk for food insecurity. There is value in understanding links between diaper and food needs, because although these forms of material hardship have the potential to exacerbate one another, they are modifiable.

## 2 Materials and methods

### 2.1 Study population

In August 2019, the Vermont WIC program sent the head of household in all 6905 participating families that accept texts from WIC (93% of all WIC households) a link to the annual online participant survey. A reminder text was sent after one week. This study was determined exempt by the University of Vermont Institutional Review Board (IRB) and Vermont Agency of Human Services IRB.

### 2.2 Measures

The survey asked about WIC implementation, food insecurity, and diaper need. Risk for food insecurity was assessed with the Hunger Vital Sign, a validated, two question screening tool based on the Household Food Security Scale (HFSS) (Hager et al., 2010). Respondents reported whether the following statements were often true, sometimes true, or never true: (1) “Within the past 12 months we worried whether our food would run out before we got money to buy more” and (2) “Within the past 12 months the food we bought just didn’t last and we didn’t have money to get more.” Following standard scoring procedures, we coded an affirmative response to either statement, as ‘at risk for food insecurity’.

Respondents were asked how many children in their household wear diapers, and those who indicated one or more children in diapers were asked about diaper need. Following Smith et al. (2013), diaper need was assessed with the question, “Do you ever feel that you do not have enough diapers to change them as often as you would like?” Those who responded “yes” were considered to report diaper need and those who responded “no” were considered to report no diaper need. Follow-up questions asked those who reported diaper need what they do when they do not have enough diapers and those who did not report diaper need what they do to have enough diapers.

Demographic factors captured through the survey included age of respondent, the household’s total length of participation in WIC, and town of residence. We categorized towns in the Burlington-South Burlington Metropolitan New England City and Town Area (NECTA) as metropolitan; all other towns were categorized as non-metropolitan (US Census Bureau, 2018).

### 2.3 Statistical analysis

We used descriptive statistics to summarize the distributions of study variables, and reviewed responses for “other” strategies to access diapers. We used Pearson’s Chi-square tests to examine the association between diaper need and risk for food insecurity, and the associations between diaper need and age of respondent, length of participation in WIC, metropolitan/non-metropolitan residence, the number of children in the household in diapers. Significance was set at α = 0.05. We performed all analyses in 2020 using IBM SPSS Statistics version 26 (IBM Corp., Armonk, NY).

Of the 761 individuals who started the survey, we excluded 73 who reported no children in the household in diapers and 187 who were missing data for diaper need or risk for food insecurity.

## 3 Results

Consistent with the largely rural composition of Vermont and the state WIC population (Vermont WIC, 2019), over two thirds of respondents (72.1%, n = 361) lived in non-metropolitan areas (Table 1). Most were over 30 (59.3%, n = 297) and had one or two children in diapers (95.6%, n = 479).

**Table 1:** Characteristics of Vermont WIC families with at least one child in diapers (n = 501), August 2019.

Variable	All participants n	Diaper need reported (n = 163) n (%)	No diaper need reported (n = 338) n (%)	*P*
At risk for food insecurity				0.000
Yes	262	120 (73.6)	142 (42.0)	
No	239	43 (26.4)	196 (58.0)	
Metropolitan area^a^				0.527
Yes	136	47 (34.6)	89 (26.5)	
No	361	114 (70.8)	247 (73.5)	
Children in the household in diapers				0.639
1 Child	371	121 (74.2)	250 (74.0)	
2 Children	108	33 (20.2)	75 (22.2)	
3 Children	22	9 (5.5)	13 (3.8)	
Age of respondent				0.480
18–24 years	72	28 (17.2)	44 (13.0)	
25–30 years	131	42 (25.8)	89 (26.3)	
31–34 years	121	34 (20.9)	87 (25.7)	
≥35 years	177	59 (36.2)	118 (34.9)	
Length of participation in WIC^b^				0.512
<6 months	56	13 (8.0)	43 (12.8)	
6 months–<1 year	75	24 (14.7)	51 (15.1)	
1–2 years	163	53 (32.5)	110 (32.6)	
4 years	81	27 (16.6)	54 (16.0)	
≥4 years	125	46 (28.2)	79 (23.4)	

a n = 497.

b n = 500.

Nearly one quarter of respondents (24.0%) reported diaper need and were at risk for food insecurity. An additional 8.6% reported diaper need but were not at risk for food insecurity and 28.3% were at risk for food insecurity but did not report diaper need. Fewer than 40% of respondents (39.1%) had neither diaper need nor risk for food insecurity. Households with diaper need were more likely to be at risk of food insecurity than households without diaper need (*p* < 0.001).

Of respondents who reported diaper need, 47.2% said that they borrow diapers or money from friends or family, 14.7% receive diapers from an agency or support organization, 59.5% stretch the diapers that they have when their supply is running short, and 12.9% use other strategies to cope. Of those who reported an adequate supply of diapers, 11.8% said that they borrow diapers or money from friends or family, 5% receive diapers from an agency or support organization, 13.9% stretch the diapers that they have, 84.6% purchase diapers with their own money, and 9.5% use some other strategies. Respondents who reported other strategies often described using cloth diapers or underwear. Some described reallocating their budget, for example, “[I] go into credit card debt for them” or “Money gets taken from food budget to buy diapers.”

## 4 Discussion

To our knowledge, this study is the first to quantify diaper need in a predominantly rural sample and among the first examine the association between diaper and food needs. Despite participating in WIC, one third of respondents with a child in diapers reported diaper need and over half were at risk for food insecurity. Disposable diapers represented a substantial financial burden for families, and those with diaper need were more likely to be at risk for food insecurity.

Prior peer-reviewed studies have quantified diaper need among low-income, urban mothers in New Haven, Connecticut using the same survey questions as the present study. The New Haven research found 27.5% of mothers with a child under 18 years and 50.3% of mothers with a child aged three years or under in diapers to report diaper need (Austin and Smith, 2017, Smith et al., 2013). The higher levels of diaper need documented in New Haven may be linked to demographic differences. Nearly 95% of Vermont’s population identify as white (US Census Bureau, 2020) compared to 9–15% of the New Haven participants (Austin and Smith, 2017, Smith et al., 2013). A survey of families utilizing a North Carolina diaper bank found a greater percentage to self-identify with a racial or ethnic minority group or speak a language other than English at home compared to the local population (Massengale et al., 2017).

Predictably, the prevalence of risk for food insecurity among our sample of Vermont WIC participants was higher than the prevalence of food insecurity previously documented among WIC participants using the US Department of Agriculture’s 18-item HFSS (Kreider et al., 2016). Prior testing against the HFSS found the Hunger Vital Sign to have a specificity of 83% indicating that 17% of families who were food secure according to the HFSS were found to be at risk for food insecurity by the Hunger Vital Sign (Hager et al., 2010). Thus, this tool may be effective in identifying families who are vulnerable to future food insecurity as well as families who are already food insecure.

The finding that diaper need is significantly associated with risk for food insecurity adds to the evidence that material hardships accumulate in families (Rodems and Shaefer, 2020) and has public health relevance. There is potential for WIC staff and pediatric providers to screen for diaper need and food insecurity and refer families to services. WIC participants are often eligible for other government assistance programs (e.g. SNAP) and continued policy and programmatic efforts are needed to streamline enrollment across programs. Further, some low-income families may benefit from referrals to local food pantries or diaper banks. Future research should explore awareness, perceptions, and use of food pantries among families with diaper need, and the role of food banks in mitigating diaper need as well as food needs. Policy interventions to reduce diaper costs or provide cash assistance to low-income families to support the purchase of diapers also could reduce diaper need. As of January 2020, 36 states charge sales tax on diapers (ranging from 2.5% to 7%) and California is the only state to provide financial assistance for diapers through an antipoverty program (the Temporary Assistance Program for Needy Families) (National Diaper Bank Network, 2020.).

In this study, examination of demographic variables with possible association to diaper need was limited to those included in the annual WIC participant survey. Although the response rate for this survey was similar to the 2018 WIC participant survey, our sample represents only 7.3% of Vermont WIC households. Response rates tend to be lower for online surveys compared to other survey formats and challenges reaching disadvantaged populations have been documented (Daikeler et al., 2019). Non-response may have biased the findings. Since most WIC participants are children, we sent the survey to the head of household, which limited our ability to compare our sample to the state WIC population. Large, diverse samples will be required to explore associations between diaper need and a broader range of demographic characteristics. The association between diaper need and risk of food insecurity found in this study needs to be replicated. Of importance, almost 30% of respondents who were at risk for food insecurity did not report diaper need. Future research should consider what practices or factors enable these families to avoid diaper need and how interventions to alleviate diaper need impact food security and child health.

## 5 Conclusions

This study calls attention to the fact that many families participating in WIC still struggle to access food and diapers and that these needs may aggravate one another, potentially leading to negative impacts on child health. Interventions to reduce diaper need may mitigate some aspects of child poverty and contribute to improvements in child nutrition and health.

## Funding

This work was funded by the University of Vermont’s Agricultural Experiment Station through the State of Vermont’s appropriation. The funder had no role in study design, data collection and analysis, decision to publish, or preparation of the manuscript.

## CRediT authorship contribution statement

**Emily H. Belarmino:** Conceptualization, Formal analysis, Writing - original draft, Funding acquisition. **Amy Malinowski:** Conceptualization, Investigation, Writing - review & editing. **Karen Flynn:** Conceptualization, Writing - review & editing.

## Declaration of Competing Interest

The authors declare that they have no known competing financial interests or personal relationships that could have appeared to influence the work reported in this paper.
